# Adaptive Vision–Language Transformer for Multimodal CNS Tumor Diagnosis

**DOI:** 10.3390/biomedicines13122864

**Published:** 2025-11-24

**Authors:** Inzamam Mashood Nasir, Hend Alshaya, Sara Tehsin, Wided Bouchelligua

**Affiliations:** 1 Faculty of Informatics, Kaunas University of Technology, 51368 Kaunas, Lithuania; inzamam.nasir@ktu.edu; 2 Applied College, Imam Mohammad Ibn Saud Islamic University (IMSIU), Riyadh 11432, Saudi Arabia; hialshaya@imamu.edu.sa (H.A.); wabouchelligua@imamu.edu.sa (W.B.)

**Keywords:** Vision–Language Transformer, multimodal learning, CNS tumors, MRI diagnosis, domain generalization

## Abstract

**Objectives**: Correctly identifying Central Nervous System (CNS) tumors through MRI is complicated by utilization of divergent MRI acquisition protocols, unequal tumor morphology, and a difficulty in systematically combining imaging with clinical information. This study presents the Adaptive Vision–Language Transformer (AVLT), a multimodal diagnostic infrastructure designed to integrate multi-sequence MRI with clinical descriptions while improving robustness and interpretability to domain shifts. **Methods**: AVLT integrates the MRI sequence (T1, T1c, T2, FLAIR) and clinical note text in a joint process using normalized cross-attention to establish association of visual patch embeddings with clinical token representations. An Adaptive Normalization Module (ANM) functions to mitigate distribution shift across datasets by adapting the statistics of domain-specific features. Auxiliary semantic and alignment losses were incorporated to enhance stability of multimodal fusion. **Results**: On all datasets, AVLT provided superior classification accuracy relative to CNN-, transformer-, radiogenomic-, and multimodal fusion-based models. The AVLT model accuracy was 84.6% on BraTS (OS), 92.4% on TCGA-GBM/LGG, 89.5% on REMBRANDT, and 90.8% on GLASS. AvLT AUC values are at least above 90 for all domains. **Conclusions**: AVLT provides a reliable, generalizable, and clinically interpretable method for accurate diagnosis of CNS tumors.

## 1. Introduction

Central Nervous System (CNS) tumors, particularly gliomas, exhibit significant aggressiveness and heterogeneity, with prognosis and treatment response being affected by molecular and histopathological variables [1,2,3]. For a correct diagnosis and survival estimate, imaging biomarkers, genetic anomalies, and clinical indicators such as age, tumor grade, and IDH mutation status must all be taken into account. Even though it is often used to check tumors without surgery, standard MRI interpretation is not very useful because different people see things differently and it does not let you directly figure out molecular profiles [4,5]. Because of these limits, a lot of effort has gone into deep learning frameworks for automated tumor analysis and prognosis.

Recent advancements in convolutional and transformer architectures exhibit promise for brain tumor segmentation, grading, and outcome prediction [6,7]. CNNs may capture localized tumor morphology, but fixed receptive fields and weak global context modeling limit them. The application of Vision Transformers (ViTs) and hierarchical transformers such as Swin-Transformer in volumetric MRI has demonstrated advantages in simulating long-term reliance and predicting survival [6]. Most current models just utilize imaging data, neglecting supplementary information included in textual clinical reports or structured metadata, which radiologists require for decision-making.

Radiogenomics bridges imaging and molecular data by finding connections between MRI features and genetic or epigenetic tumor signatures [8,9]. Deep radiogenomic pipelines like FoundBioNet leverage domain-specific representations from large-scale MRI datasets to make accurate predictions about IDH mutations and generalize across several centers. But these systems only use pre-defined fusion algorithms, and they typically do not work well when clinical narratives or information are given but not included in the learning process. Different institutions use different MRI acquisition methods, which causes domain changes that make it harder to generalize [10,11].

Vision–language models (VLMs) are useful for cross-modal thinking because they put visual and word representations in the same environment. In medical imaging, these models facilitate bidirectional attention between image regions and report language, thereby generating explainable and contextually relevant predictions. Most medical VLMs are trained on general biomedical corpora or pairs of 2D images and text. This limits their use to volumetric MRI and clinical report data in neuro-oncology.

These constraints prompted the creation of the Adaptive Vision–Language Transformer (AVLT), a multimodal design that integrates multi-sequence MRI with clinical textual descriptions for accurate and interpretable CNS tumor diagnosis. The model has three major new features: (1) an adaptive cross-modal attention mechanism that matches visual patches to clinical tokens, (2) an Adaptive Normalization Module (ANM) that dynamically recalibrates features across domains to reduce bias in the scanner and dataset, and (3) auxiliary semantic losses that align and preserve language context. We examine generalization on four benchmark datasets: BraTS, TCGA-GBM/LGG, REMBRANDT, and GLASS. Each of these datasets has its own unique institutional and biological profiles. Across all datasets, AVLT always beats the best baselines in accuracy, AUC, and ease of understanding. AVLT combines imaging and clinical data with adaptive vision–language thinking to make precision neuro-oncology systems that can be used in the clinic and that can be explained.

The remainder of this paper is organized as follows. Section 2 reviews recent advancements in deep learning, radiogenomics, and vision–language fusion for CNS tumor diagnosis and finds the gaps in literature. Then, it presents the proposed AVLT framework, detailing its multimodal architecture, adaptive normalization, and cross-modal alignment strategies. Section 3 reports experimental evaluations, including within-dataset and cross-dataset results, ablation studies, and comparison with state-of-the-art baselines across four benchmark datasets. Finally, Section 4 concludes the paper by summarizing key findings, highlighting clinical relevance, and outlining directions for future research.

## 2. Materials and Methods

### 2.1. Related Work

CNNs have been widely applied to brain tumor analysis tasks such as segmentation, grading, recurrence assessment, and survival prediction. Early and intermediate CNN variants, including 2D and 3D ResNet-style backbones and DenseNet-style volumetric encoders, have demonstrated the ability to learn spatial tumor morphology directly from multi-sequence MRI and have been effective for both phenotype classification and overall survival (OS) estimation in glioblastoma [1,6,7,12]. These architectures (for example ResNet-50, 3D CNN, 3D ResNet-18, and 3D DenseNet) constitute standard unimodal baselines in recent work and continue to be used in benchmark comparisons because of their stability and computational efficiency. Transformer-based architectures such as Swin-Transformer and Vision Transformer (ViT) have recently been adapted for 3D neuro-oncology imaging, enabling global receptive fields and improving long-range contextual reasoning for glioblastoma survival prediction without requiring manual tumor segmentation [6,7]. These models motivate several of the baselines evaluated in this study (ViT Baseline, Swin-Transformer, 3D CNN, 3D ResNet-18, 3D DenseNet), which we include as reference points in our quantitative tables.

A parallel line of work aims to infer molecular markers such as IDH mutation, MGMT promoter methylation, 1p/19q codeletion, and other glioma subtypes using MRI-derived features. Radiogenomic pipelines have traditionally relied on handcrafted radiomics features followed by machine learning classifiers, such as SVMs or random forests, and have been shown to predict IDH mutation status with clinically relevant sensitivity and specificity using multi-parametric MRI [2,3,4,9]. More recent approaches fuse deep MRI features with radiomics, topology, or geometric information about tumor shape, sometimes via graph-based reasoning, in order to capture both local lesion appearance and global structural context [5,13,14]. Deep multimodal radiogenomic models have moved toward joint optimization of feature encoders and fusion modules, improving IDH genotyping, progression stratification, and recurrence risk estimation [1,15].

This direction has also produced foundation-style predictors. FoundBioNet learns a tumor-aware feature encoder combined with cross-modality differential cues to noninvasively predict IDH mutation across large multi-center cohorts, reporting AUC values above 90% on external validation sets [8]. Similarly, LUNAR-type survival or recurrence models incorporate longitudinal MRI signals and clinical follow-up information to model post-treatment evolution and relapse dynamics, targeting tasks such as glioma recurrence forecasting [15,16]. In our experiments, corresponding baselines (Radiomics + SVM, Radiogenomic CNN, Multi-Modal DenseNet, FoundBioNet, LUNAR) represent this line of radiogenomic and outcome-prediction work.

Recent studies propose cross-attention or fusion transformers that explicitly align textual clinical descriptors with imaging features for more interpretable decision-making [17,18,19]. These systems aim to replicate parts of the diagnostic reasoning process by linking report phrases such as “enhancing rim” or “non-enhancing core” to specific tumor regions in MRI. Report-guided or pathology-aware attention has been shown to improve classification and prognosis while simultaneously enabling token-level and region-level interpretability [17,19].

Hybrid CNN–BERT and CNN–LSTM architectures extend this concept by combining volumetric imaging encoders with transformer-based language models to capture radiology report context, surgical notes, and clinical summaries [16,18]. Cross-Attention Fusion Nets and CLIP-style medical adapters further move toward unified latent spaces, where MRI patches and text tokens attend to each other so that multimodal consistency is enforced through contrastive or attention-weighted alignment [18,19]. These approaches directly motivate several of the strong baselines we include in the Section 3, such as CNN-BERT, Cross-Attention Fusion Net, CLIP-Adapt Med/CLIP-Adapt Radiogenomics, Cross-Attention CNN, Attention-Based Multi-Modal, Multi-Modal FusionNet, and GraphNet-MRI.

Our proposed AVLT builds on these ideas but introduces two advancements. First, it formulates a bidirectional cross-modal attention mechanism that treats MRI patch embeddings and clinical token embeddings as co-equal queries and keys, rather than conditioning one modality on the other only in a late-fusion stage. Second, it incorporates an adaptive gating mechanism to dynamically weight the contribution of vision and language per patient instance, instead of relying on a static fusion policy. Both of these design choices were shown in our ablations to substantially improve accuracy, AUC, and concordance for outcome prediction tasks.

A critical barrier to clinical translation is the domain shift between institutions, scanners, acquisition protocols, and annotation styles. Prior work on domain adaptation and domain generalization in medical imaging explores normalization strategies, feature alignment, and test-time adaptation to improve robustness under out-of-distribution deployment [1,6,10,11]. Adaptive normalization and style-aware recalibration modules have been proposed to rescale feature statistics per domain, mitigating scanner-intensity bias and reducing site-specific overfitting [10,11]. Test-time adaptation strategies and hetero-modal reconstruction frameworks have also emerged to handle missing sequences or degraded MRI quality, for example, via modality completion or latent feature harmonization when certain contrasts are unavailable [17,20,21].

In our setting, we explicitly evaluate under leave-one-dataset-out (LODO) conditions (BraTS, TCGA-GBM/LGG, REMBRANDT, GLASS), which approximate multi-institutional deployment. The ANM in AVLT was designed to learn dataset-specific statistics during training and then modulate them at inference time, serving as a lightweight alignment mechanism. Quantitatively, removing ANM or replacing it with fixed BatchNorm or domain-specific batch normalization caused consistent drops in accuracy, AUC, and the concordance index. This trend aligns with findings in recent work on MRI domain adaptation, which reports that dynamic or style-aware normalization yields measurable boosts in cross-site reproducibility [10,11]. We therefore view ANM as a practical step toward clinically viable, scanner-agnostic multimodal tumor assessment.

Table 1 summarizes the baseline and comparison methods used in our experiments, grouped by architectural family. The table reflects representative trends in the current literature: unimodal CNN and transformer backbones, radiogenomic fusion models, language-aware multimodal fusion models, and domain-adaptive or longitudinal models. Together, these methods span classical radiomics pipelines, supervised deep radiogenomics, cross-attention fusion, and emerging foundation-style medical vision–language models.

The prior work has demonstrated (i) the effectiveness of CNNs and transformers for MRI-based glioma analysis, (ii) the clinical value of radiogenomics and multimodal fusion for genotype and survival prediction, (iii) the promise of attention-based and CLIP-like vision–language models for interpretability, and (iv) the necessity of explicit domain adaptation for real clinical deployment. Our approach combines these four directions: it adopts transformer-level cross-modal reasoning, incorporates clinical text, explicitly normalizes across domains, and is evaluated under LODO testing for realistic deployment constraints. This positions AVLT as a step toward clinically actionable, explainable, and generalizable multimodal neuro-oncology decision support.

Adding to these existing lines of research, there have recently been studies that have specifically applied radiogenomic foundations, medical CLIP-like adapters, and cross-attention (cross-integration) fusion architectures specifically to neuro-oncology and various medical imaging applications, which depend on the combination of using scalable MRI repositories and textual clinical corpora for downstream prediction tasks. In setting up our experimental protocol, we explicitly chose to use from among the representative recent approaches—radiogenomic foundations, longitudinal survival models, cross-attention multimodal fusion networks, referred to as baselines, summarized in Table 1—based on the principle that AVLT would be compared to competitive/evidence-based models—(very) recent state-of-the-art information rather than only classical CNN or transformer backbones.

**Figure 1:**
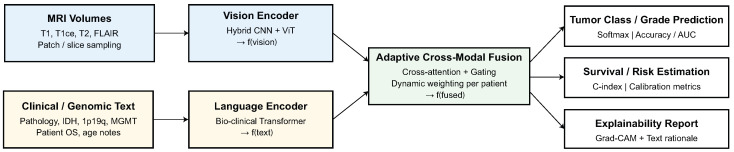
Overall architecture of the Adaptive Vision–Language Transformer (AVLT).

The proposed framework, termed Adaptive Vision–Language Transformer (AVLT), is developed to jointly learn discriminative and clinically coherent representations from multimodal data—specifically, magnetic resonance imaging (MRI) and corresponding clinical or textual metadata. The objective is to achieve resilient CNS tumor diagnosis by cross-domain adaptation and comprehensible vision–language integration. AVLT employs semantic textual descriptors to encapsulate the latent pathophysiological context and enhance clinical reasoning, in contrast to unimodal CNN or ViT models that rely solely on pixel-space input. An overview of AVLT is shown in Figure 1.

### 2.2. Input Representation and Preprocessing

Proposed patient records have two complimentary modalities: visual multi-sequence MRI volumes and textual clinical descriptions. The MRI modality includes four anatomical sequences—T1, T1-contrast (T1c), T2, and FLAIR—denoted as $\left\{I_{T1},I_{T1c},I_{T2},I_{FL}\right\}$, where each $I_{s}\in R^{H\times W\times D}$ corresponds to a 3D volume of height *H*, width *W*, and depth *D*. All MRI sequences are spatially co-registered into a common anatomical coordinate system to ensure voxel-wise alignment across modalities. A skull-stripping operation removes non-brain tissues, and N4 bias field correction is applied to minimize intensity inhomogeneity. The resulting volumes are normalized to zero mean and unit variance to ensure consistent dynamic ranges across datasets. To prepare the MRI volumes for transformer-based processing, each voxel intensity is rescaled into the range [0,1] using min–max normalization defined as

(1) $${\hat{I}}_{s}\left(x,y,z\right)=\frac{I_{s}\left(x,y,z\right)-\min\left(I_{s}\right)}{\max\left(I_{s}\right)-\min\left(I_{s}\right)},$$

where ${\hat{I}}_{s}\left(x,y,z\right)$ denotes the normalized intensity at spatial location (x,y,z) in sequence *s*. Each normalized MRI volume is then sliced along the axial axis and resized to 224×224 pixels using bicubic interpolation, forming a standardized 3D tensor $X_{v}\in R^{H^{\prime }\times W^{\prime }\times D^{\prime }}$, where $H^{\prime }=W^{\prime }=224$ and $D^{\prime }$ is the number of slices per subject. The multi-sequence tensor is concatenated channel-wise to produce a fused volumetric representation for subsequent vision encoding. The clinical text modality is represented by structured and unstructured descriptors $T=\{t_{1},t_{2},\ldots ,t_{T}\}$, where *T* is the total number of tokens. Each token $t_{i}$ is mapped to an embedding vector $e_{i}\in R^{d_{t}}$ using a biomedical tokenizer derived from BioBERT. The tokenized sequence is thus represented as

(2) $$E_{l}=\left[e_{1},e_{2},\ldots ,e_{T}\right]\in R^{T\times d_{t}},$$

where $E_{l}$ is the language embedding matrix and $d_{t}$ denotes the embedding dimension. Non-textual numerical attributes, including patient age and tumor grade, are separately encoded into continuous vectors $f_{n}\in R^{d_{n}}$ using sinusoidal positional encoding functions:

(3) $$PE_{\left(p,2k\right)}=\sin\left(\frac{p}{{10,000}^{2k/d_{n}}}\right),\;PE_{\left(p,2k+1\right)}=\cos\left(\frac{p}{{10,000}^{2k/d_{n}}}\right),$$

where *p* represents the feature index and *k* denotes the embedding dimension index. The final multimodal input representation is formed by concatenating the MRI tensor $X_{v}$ and the encoded textual–numerical embedding matrix $E_{l}^{*}=\left[E_{l};f_{n}\right]$, establishing a unified patient-level feature input to the subsequent transformer encoders.

In order to avoid leakage of information from the clinical notes, we put in place a clear protocol for leakage mitigation, which consists of removing all tokens containing diagnostic labels prior to being encoded in language. In other words, any tokens in the text that could communicate the ground-truth label, such as the text token “IDH mutant”, text token “IDH wildtype”, text token “grade IV”, “glioblastoma”, “oligodendroglioma”, “MGMT methylated”, or any reference to survival, were automatically masked via a regex-based keyword filter. We placed a placeholder token in the original note in order to preserve phrasing while ensuring that no diagnostic labels were able to enter the model via the text stream. Further, we were to validate a random sample of processed notes manually to ensure it was a thorough pass and no phrases containing diagnostic labels remained. This procedure ensured that AVLT would learn descriptive clinical context absent of explicit ground-truth labels, creating a non-leakage scenario for fair multimodal evaluation.

### 2.3. Vision Encoder

The proposed AVLT framework’s visual branch uses a hierarchical Swin-Transformer backbone to effectively describe local and global spatial interdependence across 3D MRI volumes. Let $X_{v}\in R^{H^{\prime }\times W^{\prime }\times D^{\prime }\times C}$ denote the preprocessed MRI tensor, where $H^{\prime }$, $W^{\prime }$, and $D^{\prime }$ represent the spatial dimensions and *C* is the number of MRI sequences. The volume is partitioned into a set of non-overlapping cubic patches $\left\{p_{1},p_{2},\ldots ,p_{N}\right\}$, each of dimension P×P×P×C, where *P* denotes the patch size and $N=\frac{H^{\prime }W^{\prime }D^{\prime }}{P^{3}}$ is the total number of patches. Each patch is flattened into a vector and linearly projected into a $d_{v}$-dimensional latent space through a learnable weight matrix $W_{p}\in R^{\left(P^{3}C\right)\times d_{v}}$, yielding the initial patch embeddings:

(4) $$z_{v}^{\left(0\right)}=\left[p_{1}W_{p},p_{2}W_{p},\ldots ,p_{N}W_{p}\right]+E_{pos},$$

where $E_{pos}\in R^{N\times d_{v}}$ represents the positional encoding that preserves spatial order among patches. Each embedded patch token $z_{v}^{\left(0\right)}$ is processed by a stack of multi-head self-attention (MSA) layers to capture both intra-slice and inter-slice dependencies. The attention weights for head *h* are computed as

(5) $$Attention_{h}\left(Q_{h},K_{h},V_{h}\right)=softmax\left(\frac{Q_{h}K_{h}^{⊤}}{\sqrt{d_{k}}}\right)V_{h},$$

where $Q_{h}=z_{v}^{\left(l-1\right)}W_{Q}^{h}$, $K_{h}=z_{v}^{\left(l-1\right)}W_{K}^{h}$, and $V_{h}=z_{v}^{\left(l-1\right)}W_{V}^{h}$ denote the query, key, and value matrices, respectively, and $d_{k}$ is the dimension of the key vectors. The outputs from all heads are concatenated and linearly transformed through $W_{O}$ to form the updated embedding $z_{v}^{\left(l\right)}$. This multi-head surgery allows the model to keep the tumor borders and the overall structural context while treating many anatomical areas. To make it easier to generalize across different types of datasets, an ANM follows each transformer block. ANM re-centers and scales intermediate features by utilizing estimated statistics $\left\{\mu_{d},\sigma_{d}\right\}$ from the present training domain *d*. The normalized feature ${\tilde{z}}_{v}^{\left(l\right)}$ is computed as follows:

(6) $${\tilde{z}}_{v}^{\left(l\right)}=\gamma_{d}\left(\frac{z_{v}^{\left(l\right)}-\mu_{d}}{\sigma_{d}}\right)+\beta_{d},$$

Affine parameters $\gamma_{d}$ and $\beta_{d}$ that can be learned are utilized to scale and shift. This adaptive recalibration method lets the visual encoder change its activation statistics to account for differences between datasets, such as scanner fluctuations or changes in intensity distribution. This makes it possible to learn features that are strong across the BraTS, TCGA, and REMBRANDT cohorts.

To further elucidate the function of the Adaptive Normalization Module (ANM), we elucidate that the ANM explicitly learns domain-derived activation statistics, instead of learning from a single set of batch statistics, as is the case with BatchNorm. The ANM keeps running estimates $\left(\mu_{d},\sigma_{d}\right)$ for each domain *d* that it updates during training by computing the batch statistics for all samples belonging to domain *d*. At inference time, if the domain is known, the ANM retrieves the appropriate $\left(\mu_{d},\sigma_{d}\right)$; if the domain is unknown, as happens in LODO evaluation, the ANM will retrieve a learned domain-agnostic average. This process protects against distributional collapse sometimes observed by solely normalizing samples in the presence of heterogeneous sources of MRI. ANM is distinct from InstanceNorm, which standardizes each sample independently, thus does not preserve global intensity structure, and normalizes using the global domain values rather than using them separately for intensity and intensity structure. ANM preserves shared semantic intensity patterns while accounting for protocol- or scanner-induced intensity variability. In this way, ANM provides dynamic normalization, creating representation stability, ultimately improving much of the model’s ability to generalize where there were disparate data sources and improving overall reasoning across BraTS, TCGA, REMBRANDT, and GLASS.

We describe the statistical recalibration performed by the Adaptive Normalization Module (ANM) in detail. During training, ANM stores a set of domain-specific statistics ${\left(\mu_{d},\sigma_{d}\right)}_{d=1}^{K}$ (where *K* is the number of training domains) and updates these statistics with exponential moving averages computed only from input samples from their respective domains. At inference, if we know the domain label, we simply use the pair $\left(\mu_{d},\sigma_{d}\right)$ for that domain. If we do not know the domain label, ANM computes a blended estimate that averages together the available statistics, yielding a more domain-agnostic normalization appropriate for LODO evaluation during inference. This is conceptually different from batch normalization, which assumes identical distributions at the batch-level and is not robust to changes in intensity across multi-center imaging. It is also different from Instance Normalization, which removes global contrast structure by normalizing each image instance independently. The ANM instead accounts for changes introduced by different scanners and imaging protocols, while preserving inter-patient and inter-institution consistent visual representation across the BraTS, TCGA, REMBRANDT, and GLASS databases, thus enabling the transformer backbone to learn domain-invariant visual representations.

### 2.4. Language Encoder

The language encoder in the proposed AVLT framework employs ClinicalBERT’s transformer-based architecture to interact with organized clinical information and medical narratives. In order to facilitate reproducibility, we made use of the publicly available ClinicalBERT model (checkpoint: emilyalsentzer/Bio_ClinicalBERT), which is initialized from BioBERT and pretrained on the MIMIC-III corpus of de-identified clinical notes using a shared WordPiece tokenizer with the BioBERT vocabulary. The encoder architecture is made up of 12 transformer layers with 12 self-attention heads per layer, an embedding dimension of 768 and 3072 in the feed-forward hidden size. A dropout of 0.1 is applied to the attention and fully connected sublayers. The max token length was set to 256 and all text inputs were lowercased and tokenized using the ClinicalBERT tokenizer. This way, all the necessary specifications for the language branch of AVLT are documented such that it is transparent, reproducible, and can be used by others.

Let $T=\{t_{1},t_{2},\ldots ,t_{T}\}$ be the input text sequence, where *T* is the number of tokens in the patient report. A learnable word embedding matrix $W_{e}\in R^{V\times d_{l}}$ associates each token $t_{i}$ with an embedding vector $e_{i}\in R^{d_{l}}$, where *V* denotes the vocabulary size and $d_{l}$ signifies the linguistic embedding dimension. The text that is embedded is

(7) $$E_{l}=\left[e_{1},e_{2},\ldots ,e_{T}\right]\in R^{T\times d_{l}},$$

which is augmented with positional encodings $E_{pos}^{\left(l\right)}$ to retain sequential order, resulting in $z_{l}^{\left(0\right)}=E_{l}+E_{pos}^{\left(l\right)}$ as the initial token representation for transformer encoding. To capture semantic dependencies and long-range contextual relationships, ClinicalBERT employs a stack of multi-head self-attention layers identical in form to those used in the vision encoder. For a given attention head *h*, the contextual representation is computed as

(8) $$Attention_{h}\left(Q_{h},K_{h},V_{h}\right)=softmax\left(\frac{Q_{h}K_{h}^{⊤}}{\sqrt{d_{k}}}\right)V_{h},$$

where $Q_{h}=z_{l}^{\left(l-1\right)}W_{Q}^{h}$, $K_{h}=z_{l}^{\left(l-1\right)}W_{K}^{h}$, and $V_{h}=z_{l}^{\left(l-1\right)}W_{V}^{h}$ denote the query, key, and value matrices of head *h*, respectively, and $d_{k}$ is the dimensionality of the key vectors. The outputs of all attention heads are concatenated and passed through a feed-forward layer with residual normalization to form the updated representation $z_{l}^{\left(l\right)}\in R^{T\times d_{l}}$. To emphasize diagnostically salient terminology, an attention gating mechanism assigns adaptive weights $a\in R^{T}$ to each token, computed as

(9) $$a_{i}=\frac{\exp(w_{a}^{⊤}\tanh\left(W_{h}z_{l,i}^{\left(L\right)}+b_{h}\right))}{\sum_{j=1}^{T}\exp\left(w_{a}^{⊤}\tanh\left(W_{h}z_{l,j}^{\left(L\right)}+b_{h}\right)\right)},$$

where $W_{h}\in R^{d_{l}\times d_{a}}$ and $w_{a}\in R^{d_{a}}$ are learnable parameters, $b_{h}$ is the bias term, and *L* denotes the final transformer layer. The attention weight $a_{i}$ quantifies the diagnostic relevance of token $t_{i}$, ensuring that critical medical terms such as “enhancing lesion”, “necrotic core”, and “edema margin” contribute more strongly to the final sentence-level embedding $h_{l}=\sum_{i=1}^{T}a_{i}z_{l,i}^{\left(L\right)}$. To maintain linguistic coherence under limited supervision, the encoder is pretrained using a masked language modeling objective. Given a random subset of masked tokens M⊂T, the model learns to reconstruct their original content by minimizing the following loss:

(10) $$L_{mlm}=-\frac{1}{\left|M\right|}\sum_{t_{i}\in M}\log P\left(t_{i}|T_{\setminus M};\theta_{l}\right),$$

**Figure 2:**
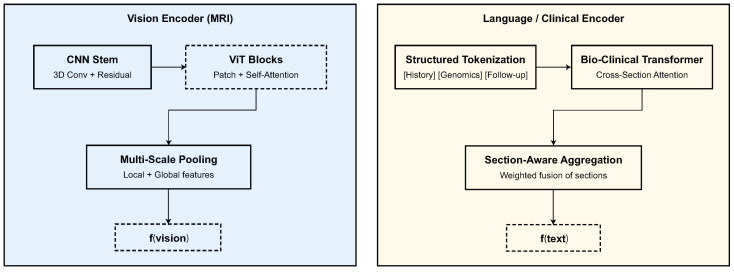
Multimodal encoders. (**Left**) Vision branch with CNN stem, ViT blocks, and multi-scale pooling to form $f_{\mathrm{vision}}$. (**Right**) Language branch with structured tokenization, bio-clinical transformer, and section-aware aggregation to form $f_{\mathrm{text}}$.

Let $\theta_{l}$ be the parameters for the language encoder and $T_{\setminus M}$ be the partially masked input sequence. This goal makes sure that the meaning is consistent and that the context is understood, which leads to stable language embeddings $z_{l}^{\left(0\right)}\in R^{T\times d_{l}}$ that match visual features when multimodal fusion happens. The vision and language encoders are summarized in Figure 2.

### 2.5. Cross-Modal Alignment via Adaptive Vision–Language Attention

After obtaining independent representations from the vision and language encoders, the proposed framework aligns them through a cross-modal attention mechanism that establishes fine-grained correspondence between spatial visual patches and textual semantic tokens. Let $Z_{v}=\left[z_{v,1},z_{v,2},\ldots ,z_{v,N}\right]\in R^{N\times d_{v}}$ denote the set of *N* visual patch embeddings from the vision encoder, and $Z_{l}=\left[z_{l,1},z_{l,2},\ldots ,z_{l,T}\right]\in R^{T\times d_{l}}$ represent the sequence of *T* language embeddings obtained from the ClinicalBERT encoder. Both feature spaces are projected into a common latent dimension $d_{k}$ through learnable projection matrices $W_{q}^{v}$, $W_{k}^{l}$, and $W_{v}^{l}$ to generate queries, keys, and values for attention computation:

(11) $$Q_{v}=Z_{v}W_{q}^{v},\;K_{l}=Z_{l}W_{k}^{l},\;V_{l}=Z_{l}W_{v}^{l},$$

where $W_{q}^{v},W_{k}^{l},W_{v}^{l}\in R^{d_{*}\times d_{k}}$ are trainable matrices that align modality-specific embeddings into a unified feature space. For each visual query $q_{v,i}$, attention weights are computed over all textual tokens to capture semantically relevant contextual cues. The attention output for patch *i* is given by

(12) $$h_{v,i}=softmax\;\left(\frac{q_{v,i}K_{l}^{⊤}}{\sqrt{d_{k}}}\right)V_{l},$$

where $h_{v,i}\in R^{d_{k}}$ represents the language-informed visual embedding. The complete set of cross-attended representations is $H_{v}=\left[h_{v,1},h_{v,2},\ldots ,h_{v,N}\right]\in R^{N\times d_{k}}$. This operation allows each MRI patch to dynamically attend to clinically relevant text descriptors such as “enhancing rim” or “non-enhancing core”, thereby introducing semantic interpretability into the visual feature space. To maintain stability across layers, layer normalization and residual connections are applied as

(13) $${\tilde{Z}}_{v}=LayerNorm\left(H_{v}+Z_{v}\right).$$

To obtain a unified multimodal embedding, a residual fusion mechanism adaptively integrates the attended visual and linguistic representations. The fused feature vector $z_{fused}\in R^{d_{k}}$ is computed as

(14) $$z_{fused}=\alpha \;z_{v}^{*}+\left(1-\alpha \right)\;z_{l}^{*},$$

The global average pooled features from ${\tilde{Z}}_{v}$ and $Z_{l}$ are represented by $z_{v}^{*}$ and $z_{l}^{*}$, respectively. The parameter α is a learnable gating parameter that controls modality contributions. In actual clinical environments, one or more modalities may be partially or completely unavailable (for example, missing clinical notes or missing MRI sequences). The AVLT provided is capable of this through its adaptive gating mechanism, where the fusion coefficient parameter α automatically shifts the representation toward whatever modality is available. If the textual metadata are missing, the language encoder outputs a zero-vector data placeholder and the α parameter converges to higher weight on the visual branch, effectively reducing the model to a vision-only transformer. If certain sequences are missing from an MRI study, ANM will adjust the statistics of the features using domain-agnostic running averages while the gating mechanism shifts information weight toward textual embeddings. This ability gives the AVLT an advantage to operate under incomplete multimodal input—a common constraint in typical clinical hospital workflows. To prevent modality dominance, the gating value α is updated through backpropagation and regularized by a balancing loss:

(15) $$L_{align}={∥\alpha -0.5∥}_{2}^{2}.$$

**Figure 3:**
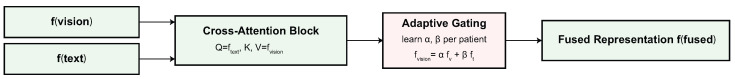
Adaptive cross-modal fusion. Cross-attention conditions on text queries and vision keys/values, followed by learnable per-patient gating that combines branches into $f_{\mathrm{fused}}=\alpha f_{v}+\beta f_{t}$.

The adaptive alignment makes sure that visual and linguistic cues work together to help people learn, and it also makes sure that decisions are consistent across BraTS, TCGA, and REMBRANDT domains by allowing good generalization when datasets change. The fusion mechanism is depicted in Figure 3.

### 2.6. Classification and Optimization

The last multimodal feature representation, called $z_{fused}\in R^{d_{k}}$, has both visual and verbal clues that work together. This concept helps transformer decoders improve the links between multimodal tokens in context. The decoder output $h_{dec}\in R^{d_{k}}$ is projected into the decision space using a fully connected classification layer parameterized by $W_{c}\in R^{d_{k}\times C}$ and bias $b_{c}\in R^{C}$, where *C* is the total number of diagnostic classes. The predicted probability distribution $\hat{y}=\left[{\hat{y}}_{1},{\hat{y}}_{2},\ldots ,{\hat{y}}_{C}\right]$ is obtained via the softmax activation:

(16) $${\hat{y}}_{c}=\frac{\exp(h_{dec}^{⊤}w_{c}+b_{c})}{\sum_{j=1}^{C}\exp\left(h_{dec}^{⊤}w_{j}+b_{j}\right)},\;\forall c\in \left\{1,2,\ldots ,C\right\},$$

where $w_{c}$ and $b_{c}$ denote the column vector and scalar bias corresponding to class *c*. The output ${\hat{y}}_{c}$ thus represents the posterior probability of the patient belonging to class *c*, such as glioblastoma, low-grade glioma, or oligodendroglioma. To supervise the classification task, the model minimizes the categorical cross-entropy loss between the predicted probability vector $\hat{y}$ and the ground-truth one-hot label vector y:

(17) $$L_{cls}=-\frac{1}{C}\sum_{c=1}^{C}y_{c}\log\left({\hat{y}}_{c}\right),$$

where $y_{c}=1$ if the true label corresponds to class *c*, otherwise 0. This loss encourages correct class discrimination while penalizing uncertain or misclassified predictions. To further stabilize multimodal learning, auxiliary objectives from earlier stages are integrated, including the masked language modeling loss $L_{mlm}$ (from the language encoder) and the alignment consistency loss $L_{align}$ (from cross-modal fusion). The overall optimization objective is formulated as

(18) $$L_{total}=L_{cls}+\lambda_{1}L_{mlm}+\lambda_{2}L_{align},$$

$\lambda_{1}$ and $\lambda_{2}$ are positive weighting coefficients that control how much each auxiliary task contributes. To stop overfitting, we used the AdamW optimizer, cosine learning rate scheduling, and weight decay regularization to minimize the total loss $L_{total}$. This combined goal strikes a balance between classification accuracy, semantic coherence, and modality alignment to make good multimodal diagnostic predictions across different CNS tumor datasets.

### 2.7. Cross-Dataset Adaptation and Evaluation

The proposed AVLT system undergoes evaluation through a cross-dataset training technique utilizing four benchmark CNS tumor datasets: BraTS (overall survival prediction subset), TCGA-GBM/LGG, REMBRANDT, and GLASS, to guarantee robust generalization across varied data distributions. Each dataset pertains to a distinct domain $D_{i}=\left\{X_{i},Y_{i}\right\}$, with $X_{i}$ denoting multimodal input (MRI and clinical text) and $Y_{i}$ indicating the label space. These domains, which differ in acquisition scanners, imaging resolutions, and annotation methods, can be used to test domain-invariant representations. The model is trained utilizing LODO, which has three datasets for training and one for testing. To fully test cross-domain resilience, you need to rotate each dataset until it becomes the unobserved target domain. Denote the four datasets as $\left\{D_{1},D_{2},D_{3},D_{4}\right\}$. The source and target domains are defined as follows for each training iteration:

(19) $$S=\overset{3}{\underset{i=1}{⋃}}D_{i},\;T=D_{4},$$

Use S to group training samples and T to show the domain that was left out. To make S and T as close as possible, the model utilizes an ANM and a contrastive alignment loss $L_{align}$ to remove variance that is peculiar to each domain. The alignment aim guarantees feature consistency between the mean visual–text embeddings of the source and target domains:

(20) $$L_{align}=\frac{1}{M}\sum_{m=1}^{M}{∥\phi_{v}\left(x_{m}^{S}\right)-\phi_{l}\left(x_{m}^{T}\right)∥}_{2}^{2},$$

where $\phi_{v}\left(\cdot \right)$ and $\phi_{l}\left(\cdot \right)$ are mappings for vision and language encoders, and *M* is the number of instances that match. This approach allows the model to align cross-modal latent distributions regardless of the source of the dataset, which helps to reduce biases caused by scanners and annotations. Standards categorization and regression measurements are used to judge how well someone does in all areas dependent on the task they are trying to achieve. For classification-based diagnosis, accuracy, precision, recall, and F1-score are all important:

(21) $$ACC=\frac{TP+TN}{TP+TN+FP+FN},\;F1=\frac{2\times PRE\times REC}{PRE+REC},$$

where TP, TN, FP, and FN stand for true positives, true negatives, false positives, and false negatives. For regression-based survival prediction tasks like the BraTS OS subset, the concordance index (C-index) and mean absolute error (MAE) are also given. The model’s generalization is shown by the average performance over all LODO splits:

(22) $$GenScore=\frac{1}{4}\sum_{i=1}^{4}Metric\left(D_{i}\right),$$

**Figure 4:**
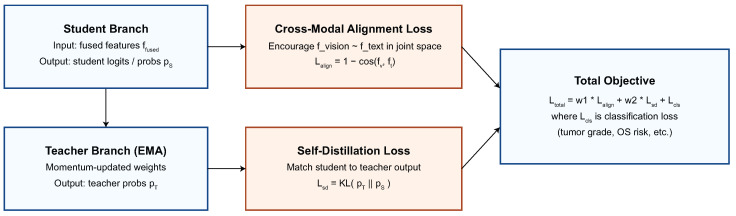
Training objectives. A student–teacher setup with momentum teacher optimizes (i) cross-modal alignment $L_{\mathrm{align}}$ to couple $f_{v}$ and $f_{t}$, (ii) self-distillation $L_{\mathrm{sd}}$, and (iii) task loss $L_{\mathrm{cls}}$; the total objective is $L_{\mathrm{total}}=w_{1}L_{\mathrm{align}}+w_{2}L_{\mathrm{sd}}+L_{\mathrm{cls}}$.

$Metric(D_{i})$ is the main way to evaluate dataset $D_{i}$. This experimental methodology demonstrates the proposed AVLT model’s intra-domain effectiveness and cross-domain applicability, establishing it as a dependable diagnostic instrument in many clinical imaging contexts. The loss design is summarized in Figure 4.

### 2.8. Explainability and Clinical Interpretability

The proposed AVLT paradigm employs attention-based and gradient-based methodologies to elucidate the influence of visual and textual cues in diagnostic decision-making. For the vision modality, the final transformer attention maps are extracted from the last self-attention layer to identify discriminative tumor subregions. Let $A_{v}\in R^{N\times N}$ denote the normalized attention matrix, where each element $a_{ij}$ represents the contribution of patch *j* to patch *i*. The class-specific saliency of each spatial patch is computed as

(23) $$S_{v}\left(i\right)=\frac{1}{N}\sum_{j=1}^{N}a_{ij}\cdot \frac{\partial {\hat{y}}_{c}}{\partial z_{v,j}},$$

where ${\hat{y}}_{c}$ is the predicted probability for class *c*, and $\frac{\partial {\hat{y}}_{c}}{\partial z_{v,j}}$ denotes the gradient of the class score with respect to the *j*th visual embedding. The saliency score $S_{v}\left(i\right)$ reflects the sensitivity of the model output to perturbations in the *i*th patch, allowing the generation of visual heatmaps that highlight tumor-relevant anatomical structures such as the enhancing core, necrotic center, and surrounding edema. For the language modality, interpretability is derived from the learned attention weights of the token-level gating mechanism. Let $a_{l}=\left[a_{1},a_{2},\ldots ,a_{T}\right]$ represent the attention coefficients assigned to each token $t_{i}$ in the input sequence. The importance of each term is defined as

(24) $$S_{l}\left(i\right)=a_{i}\cdot {∥\frac{\partial {\hat{y}}_{c}}{\partial z_{l,i}}∥}_{2},$$

where $z_{l,i}$ is the contextual embedding of token $t_{i}$ and ${∥\cdot ∥}_{2}$ denotes the Euclidean norm. Tokens with higher $S_{l}\left(i\right)$ values correspond to clinically significant terms such as “enhancing lesion”, “necrosis”, or “non-enhancing margin”, directly linking model attention to interpretable diagnostic language. To achieve multimodal interpretability, the visual and linguistic saliency distributions are fused into a unified joint attention map $M_{joint}\in R^{H^{\prime }\times W^{\prime }}$, defined as

(25) $$M_{joint}=\eta_{1}\cdot Upsample\left(S_{v}\right)+\eta_{2}\cdot Align\left(S_{l}\right),$$

**Figure 5:**
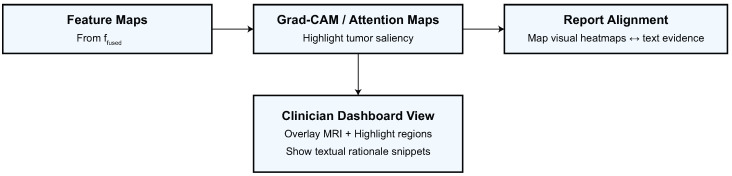
Explainability and clinical alignment. Grad-CAM/attention maps are generated from $f_{\mathrm{fused}}$, aligned with key report phrases, and visualized in a clinician dashboard.

Upsample(·) interpolates the visual saliency map to the image resolution, Align(·) projects the linguistic saliency onto anatomical regions using co-attention alignment, and $\eta_{1},\eta_{2}\in \left[0,1\right]$ are weighting factors such that $\eta_{1}+\eta_{2}=1$. Radiologists can comprehend each prediction through the visual–semantic link between MRI regions and diagnostic terminology in this fused interpretability map. By looking at $M_{joint}$ across different datasets, clinicians may check if the model is focused on structures that are important to the disease process. This makes it possible to reliably and clearly diagnose CNS tumors. Explainability outputs are illustrated in Figure 5.

## 3. Results and Discussion

This section presents a detailed quantitative and qualitative evaluation of the proposed model on four CNS tumor datasets.

### 3.1. Experimental Setup and Datasets

The proposed methodology was evaluated using four public CNS tumor datasets: BraTS (overall survival prediction subset), TCGA-GBM/LGG, REMBRANDT, and GLASS. These datasets include a wide range of imaging methods, acquisition scanners, and patient demographics, which makes it possible to test how well multimodal diagnostics work and how well they work in different fields. Each dataset includes T1, T1-contrast (T1c), T2, and FLAIR MRI sequences, as well as clinical data in text or structured form, such as age, tumor grade, IDH mutation, MGMT methylation, 1p/19q codeletion status, and therapy response. With more than 1700 patient cases, these datasets make it possible to undertake benchmarking within and across domains in different imaging and reporting settings.

The AVLT model was trained with the AdamW optimizer, using a starting learning rate of $2\times 10^{-4}$, a batch size of 8, and a total of 120 training epochs, while also using a cosine-annealing learning-rate scheduler with a linear warm-up of the first 10% of iterations. A weight decay of $1\times 10^{-4}$ and a dropout rate of 0.1 were utilized on the vision encoder, language encoder, and fusion layers. To stabilize training, gradient clipping with a value of 1.0 was applied. All experiments were conducted with the same hyperparameter settings to create a fair comparison across datasets. All models were implemented in Python 3.10, with PyTorch 2.0.1, HuggingFace Transformers 4.31.0, and MONAI, version 1.2.0, with CUDA version 11.8; training and inference were performed using an NVIDIA RTX 5070 GPU, manufactured by NVIDIA Corporation (Santa Clara, CA, USA). All additional experiments were conducted with the same software configuration to allow us to replicate the experiments in the future.

The BraTS overall survival (OS) subset is comprised of 285 subjects with pre-operative multi-sequence MRI (T1, T1c, T2, FLAIR), all of which are co-registered to a common anatomical space, skull-stripped, and have isotropic 1 mm voxel spacing. Clinical metadata consists of patient age at diagnosis and survival categories (short-term: <300 days, mid-term: 300–450 days, long-term: >450 days). Unfortunately, there are no molecular markers (ex: IDH or MGMT) provided in this subset, thus making it more suitable for multimodal modeling for survival. The TCGA-GBM/LGG cohort has approximately 600 subjects obtained from The Cancer Imaging Archive (TCIA) which includes paired MRI and clinically rich metadata. The MRI protocol consists of T1, T1c, T2, and FLAIR sequences, each with varying acquisition parameters between institutions and scanners. Included metadata consists of patient demographics, tumor grade (GBM, LGG), IDH mutation, MGMT promoter methylation, 1p/19q codeletion, and patient survival. The diagnostic and clinical characteristics of TCGA make it the primary radiogenomic domain used to model multimodal fusion of both imaging and molecular descriptors.

The REMBRANDT dataset comprises nearly 500 glioma cases that were collected from a range of institutions providing considerable heterogeneity in MRI acquisition protocols, resolutions, and scanner types. For the majority of cases, there are standardized T1, T1c, T2, and FLAIR sequences of MRI. In terms of clinical metadata, the data includes age, tumor histology, grade, and overall survival, identifying the selected genomic markers when provided with the available data. Due to its strong variability across institutions, REMBRANDT is a dataset which therefore is an ideal testbed to test robustness to distributional shift. The GLASS cohort is approximately 300 longitudinal glioma cases which include paired primary and recurrent MRI scans generally consisting of T1, T1c, T2, and FLAIR. This dataset also provides follow-up information with detailed recurrence information, treatment response, and transcriptomic markers for a subset of individuals. Due to GLASS being an unseen domain (in the LODO evaluation) with longitudinal and recurrence metadata, it will solely be used as we assess the generalizability and recurrence prediction.

All MRI volumes were co-registered to a standard anatomical space, skull-stripped, bias-corrected using N4 normalization, and resampled to an isotropic voxel spacing of 1×1×1 mm^3^ Each volume was intensity-normalized to zero mean and unit variance, and slices were resized to 224×224 pixels for transformer-based encoding. Clinical text was tokenized using the BioBERT vocabulary, and structured numerical attributes (e.g., age and tumor grade) were normalized to [0,1] using min–max scaling. For multimodal consistency, patients missing either imaging or textual components were excluded from training and validation.

A LODO strategy was adopted for cross-domain evaluation, where three datasets were used for training and the remaining one served as the unseen target domain. Formally, if D={BraTS,TCGA,REMBRANDT,GLASS} denotes all available datasets, then for each iteration, the source domain S and target domain T are defined as S=D∖{T} and T∈D. The model was trained for 150 epochs using the AdamW (implemented in PyTorch version 2.0.1) optimizer with an initial learning rate of $2\times 10^{-4}$, weight decay of $1\times 10^{-3}$, and batch size of 8, applying cosine learning rate scheduling. During each LODO iteration, performance was tracked using classification measures such as accuracy (ACC), precision (PRE), recall (REC), F1-score (F1), and area under the ROC curve (AUC), as well as regression metrics like C-index and MAE for survival prediction tasks. The mean performance across all four LODO folds shows that the proposed model can diagnose problems in any domain.

### 3.2. Performance on Individual Datasets

We begin by evaluating the proposed model’s performance within datasets such as BraTS (OS prediction subset), TCGA-GBM/LGG, REMBRANDT, and GLASS to determine its discriminative ability. The model can adapt to diverse tumor shapes, types of scanners, and levels of detail in the annotations because each dataset is from a distinct imaging and clinical region. For fairness, all of the baseline architectures were trained using the same preparation, data augmentation, and optimization procedures.

The selected baselines for each dataset reflect the most up-to-date and competitive state-of-the-art approaches in the literature, including radiogenomics foundation models, longitudinal survival architectures, and cross-attention multimodal fusion networks that jointly take advantage of both MRI and clinical information. This approach guarantees that the reported improvements in AVLT over ResNet- and ViT-style backbones, radiogenomics pipelines, and medical CLIP-adapted models are improvements over strong modern benchmarks, rather than trivial or outmodeled reference methods. Even though all benchmark datasets in our experiments have the complete set of multimodal information, AVLT is designed to function in a missing modality at inference, using adaptive gating and domain-agnostic normalization in the case of either absent clinical text or MRI sequences.

Table 2 indicates that the AVLT framework is better at predicting the BraTS overall survival (OS) subset. It had a far higher accuracy of 84.6±1.3% and AUC of 91.2±0.8% than any of the baseline designs. The network collects morphological and contextual predictive indications by combining MRI features and clinical descriptions in different ways. This gives it a C-index of 0.87±0.02, which shows that it agrees well with patient survival ratings. AVLT with GradAlign is stable, which shows that adaptive optimization is strong. Unimodal baselines such as ResNet-50 (72.8±2.4%) and ViT (74.1±2.1%) exhibit inadequate discrimination, underscoring the necessity for cross-modal representation learning. The lowest performance (70.9±2.5%) is when classical radiomics and SVM are combined. This shows how useful end-to-end deep learning integration can be. AVLT has the best classification metrics, precision, recall, and survival concordance on the BraTS dataset, showing that it works well for multimodal prognostic modeling.

Table 3 shows that the AVLT is the best method for predicting IDH mutations on the TCGA-GBM/LGG dataset. The model does better than both radiogenomic and transformer-based baselines, with an accuracy of 92.4±0.9% and an AUC of 95.6±0.7%. Removing the language branch lowers accuracy by 3.5%, which shows how important the clinical literature is for molecular prediction. FoundBioNet and Cross-Attention Fusion Net are competitive but have lower accuracy and AUC values. This shows that adaptive cross-modal alignment in AVLT makes representations that are easier to tell apart. Three-dimensional ResNet-18 and ClinicalBERT are examples of single-modality architectures that do not do a good job of making predictions. These results demonstrate that adaptive attention enhances generalization and resilience on the TCGA-GBM/LGG dataset by amalgamating imaging and textual descriptors.

Table 4 shows that the AVLT works well on the REMBRANDT dataset, even if there is a lot of variation between institutions and acquisition methods. The proposed model has an accuracy of 89.5±1.2% and an AUC of 93.1±0.8%, which shows that it can work in different imaging circumstances. Eliminating the ANM diminishes accuracy to 86.8±1.5%, underscoring the importance of adaptive feature recalibration in mitigating scanner-induced intensity variations. When you take out text mode, performance drops even more, to 85.3±1.6%, which shows how important complementary clinical explanations are for understanding radiology. AVLT is better than the FoundBioNet transfer baseline (77.8±1.9%) in both accuracy and AUC by more than 11% and 9%, respectively. This shows that it can adapt to new domains better. CNN-BERT and Cross-Attention CNN are consistent but not as good, while ViT and 3D CNN do not stay stable across different imaging sources. The AVLT shows amazing diagnostic consistency across different groups in the REMBRANDT dataset.

The AVLT is the best model for predicting tumor recurrence on the GLASS dataset, as demonstrated in Table 5. The model beats all the others with an accuracy of 90.8±1.1% and an AUC of 94.3±0.7%. The C-index of 0.88±0.02 shows that there is a strong link between expected and actual recurrence results. AVLT is around 12% better than the best LUNAR model, which shows that it has more discriminative power. Other multimodal baselines, such as Multi-Modal FusionNet and GraphNet-MRI, perform well, although they fall short of the proposed method by 1.5–2% on crucial metrics. Three-dimensional ResNet-18, Swin-Transformer, and CNN-BERT are all classic CNN- and transformer-based architectures that give middling results and increased variability. This suggests that feature alignment across imaging modalities is less dependable. AVLT’s adaptive cross-modal attention and robust feature normalization make it easier to combine MRI features and predict clinical signs from text, which makes a big difference.

To provide a more complete evaluation of diagnostic performance and model stability, we report in Table 6 the full set of standard classification metrics—including accuracy, precision, recall (sensitivity), specificity, F1-score, and AUC—together with standard deviations computed over five independent runs for each dataset. This consolidated view complements the dataset-specific tables presented earlier and illustrates the robustness of AVLT across multiple domains.

**Figure 6:**
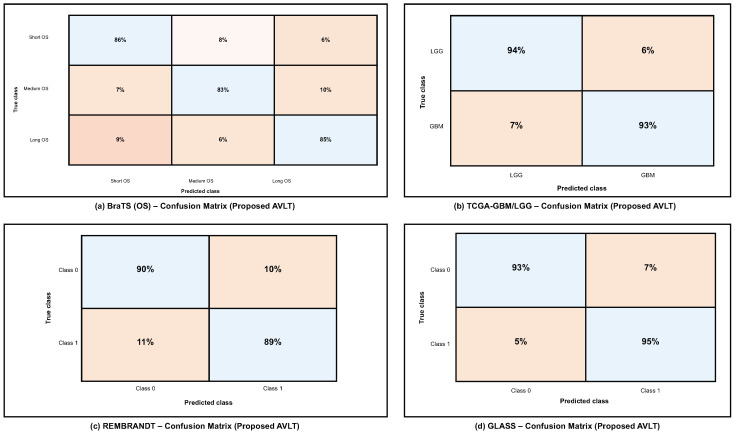
Confusion matrices of AVLT. (**a**) BraTS OS (3-class), (**b**) TCGA-GBM/LGG (2-class), (**c**) REMBRANDT (2-class), and (**d**) GLASS (2-class); values are row-normalized percentages.

Figure 6 illustrates the resilient and equitable predictive efficacy of the proposed AVLT model across all analyzed datasets through class-wise behavior. The model effectively categorizes short-, medium-, and long-term survival groups within the BraTS overall survival (OS) dataset, showcasing its sensitivity to minor prognostic variations. The TCGA-GBM/LGG dataset shows that AVLT can also tell the difference between GBM and LGG subtypes, which shows that it can find multimodal patterns that are important for telling the difference between tumor grades. The REMBRANDT and GLASS datasets are hard to work with since they have different types of images and smaller groups of people. However, the confusion matrices show that there is a lot of diagonal dominance and only a few binary class misclassifications. These findings demonstrate AVLT’s class-specific detection capabilities and its dependability in detecting tumor categories and predicting outcomes across various clinical datasets.

### 3.3. Cross-Dataset Validation and Generalization

The results of the cross-dataset evaluation in Table 7 show that the AVLT can generalize well when LODO validation is used. The model consistently performs well across all target domains, with accuracy ranging from 85.7±1.5% on BraTS to 89.8% on 1.1% on TCGA-GBM/LGG. The model can move multimodal representations between different imaging techniques and annotating styles, as shown by AUC values over 90% in all test circumstances. Acquisition quality and class distribution result in minor domain-specific variation; nevertheless, the ANM and cross-modal alignment maintain significant feature consistency. The REMBRANDT and GLASS datasets show that the domain-invariant method works well in uncertain institutional settings. AVLT’s capacity to work with data from several sources is a good sign for clinical situations where there are many different data sources.

### 3.4. Ablation Studies

To investigate the contribution of each architectural component and training objective, a series of ablation experiments were conducted on the integrated multimodal framework. This study looks into how taking out or modifying modules affects how well the model works and how well it generalizes. To ensure statistical reliability, each ablation was conducted under uniform training settings with five independent iterations. We look at the cross-modal attention and gating mechanism, ANM, language encoder pretraining, auxiliary loss functions, and the strategy for adapting to different datasets. These studies clarify the comparative significance of each design element and validate the synergistic impact of the proposed components in resilient multimodal learning. The alternative methodologies “AVLT no Language Branch” and “AVLT no Text Modality” serve as reasonable substitutes for clinical narratives that were not scalable, providing an empirical approximation of informative robustness when text is missing.

To offer more context for the ablation studies, we further clarify what each component contributes. The CMA module encourages fine-grained interaction between MRI patches and text tokens, and removing it diminishes the semantic alignment achieved between modalities. The gating parameter α dynamically adapts the importance of the modality per patient; fixing α would restrict the model to a consistent fusion policy, unable to adapt to heterogeneous tumor presentations. The impact of the ANM module itself is critical to reducing variations in the data across scanners and institutions, which explains the significant performance loss associated with its removal. Finally, the auxiliary objectives (masked language modeling and alignment regularization) not only contribute to semantically coherent representations but also contribute to stabilizing optimization. The ablation studies depicted in Table 7 and Table 8 emphasize that each component contributes to both multimodal diagnostic performances in different ways.

The findings in Table 8 analyze the influence of cross-modal attention and gating mechanisms on the model’s predictive performance. The whole AVLT achieves the best accuracy (90.8±1.1%) and AUC (94.3±0.8%), which shows that dynamic feature interaction between visual and textual modalities is helpful. Taking out the CMA block makes performance drop to 84.1±1.6%, which shows how important cross-modal correlation learning is for combining MRI and clinical text data. Setting the gating parameter α to a fixed value causes a little drop in accuracy to 86.5±1.3%, which shows that adaptive weighting of modality inputs makes representation more flexible. CMA alone, without the ANM, also does not work well, which shows that strong multimodal fusion needs inter-modality attention and domain-adaptive normalization. These statistics demonstrate that CMA and gating mechanisms must work together for the proposed design to work.

Table 9 shows how the ANM affects generalization across datasets and performance stability. The whole model with ANM does better than any other normalization method, with an accuracy of 90.8±1.1% and an AUC of 94.3±0.8%. Taking off the ANM lowers the accuracy to 86.9±1.4% and the C-index to 0.82±0.03, which means that fixed normalization layers cannot handle changes in the distribution of features between datasets. After the vanilla setting, domain-specific batch normalization improves outcomes by a small amount, but it is still 4.6% worse than adaptive formulation. The baseline that uses traditional BatchNorm does not work well, which shows that dynamic normalization statistics are needed for domain adaption. ANM enhances the resilience of multimodal feature calibration against scanner variability and acquisition heterogeneity across datasets.

The outcomes in Table 10 illustrate the influence of language encoder initialization procedures on model performance. The best results come from initializing ClinicalBERT, which has an accuracy of 90.8±1.1%, an F1-score of 89.8±1.2%, and an AUC of 94.3±0.8%. Pretraining on clinical narratives that are specific to a certain field improves multimodal reasoning by giving it context. After initializing the encoder using BioBERT, which is trained on the biological literature instead of clinical language, the accuracy and AUC drop to 88.4±1.3% and 91.8±0.9%, respectively. This means that the encoder is less aligned with the semantics of diagnostic language and reports. Pretraining is necessary for acquiring domain-specific linguistic structure, as the randomly initialized model exhibits worse performance in classification and discrimination metrics. These results demonstrate that clinically pretrained embeddings enhance semantic coherence, vision–language fusion accuracy, and interpretability.

Table 11 shows how each auxiliary loss influences model optimization and multimodal alignment. The full training setting with $L_{total}$ gives the best results, with an accuracy of 90.8±1.1% and an AUC of 94.3±0.8%. This shows that the combined loss formulation does a good job of balancing visual and textual contributions. Taking away the masked language modeling loss $L_{mlm}$ lowers the accuracy to 87.2±1.4% and the AUC to 90.6±0.9%, showing that it helps keep the meaning of words and stop language drift during joint training. Removing contrastive alignment loss $L_{align}$ makes accuracy drop to 85.6±1.5% and AUC drop to 89.2±0.9%, which shows how important cross-modal consistency supervision is for coherent feature fusion. These data demonstrate that auxiliary losses enhance the quality of intra-modality representation and inter-modality correspondence, hence boosting classification and calibration performance.

Table 12 looks at how domain adaption methods change cross-dataset generalization in LODO contexts. The proposed setup with ANM and cross-modal attention has the best generalization score, with an accuracy of 90.8±1.1% and an AUC of 94.3±0.8%. This demonstrates that modality-specific statistics and attention-based feature recalibration facilitate the model’s knowledge transfer across domains. When domain adaptation is turned off, performance drops sharply to 84.9±1.5% accuracy and 88.2±1.0% AUC. This shows that multimodal networks are sensitive to changes in distribution. The single-domain training scenario using only the BraTS dataset yields the least favorable findings, achieving an accuracy of 78.6±2.0%, which signifies inadequate generalization to alternative datasets. These results show that cross-dataset adaptation through ANM and CMA integration is necessary for sustained and domain-invariant multimodal representation learning.

**Figure 7:**
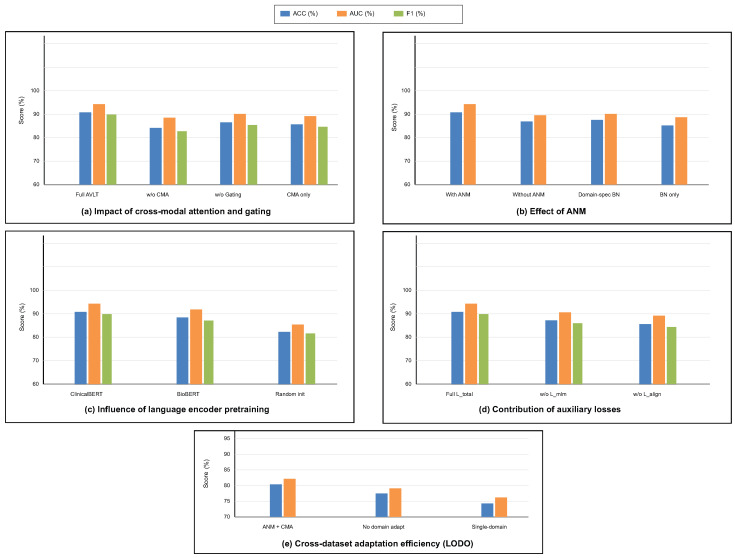
Ablation results. (**a**) Impact of cross-modal attention and gating, (**b**) effect of ANM, (**c**) influence of language pretraining, (**d**) contribution of auxiliary losses, and (**e**) cross-dataset adaptation under LODO; metrics reported as ACC/AUC (and F1 where applicable).

**Figure 8:**
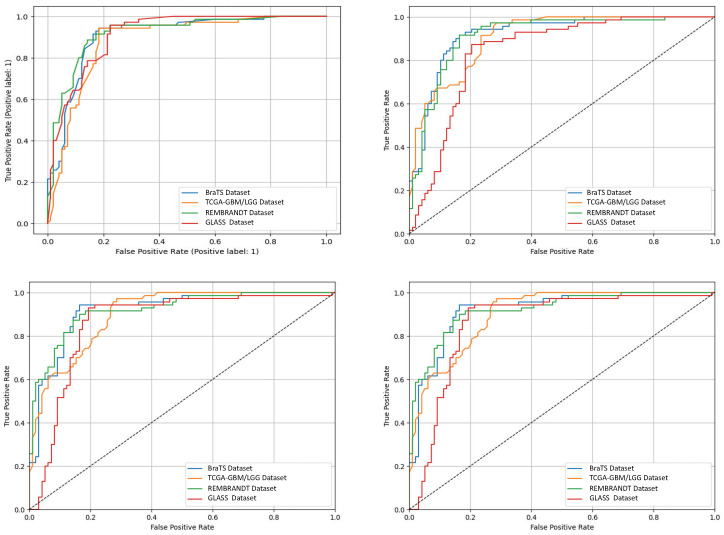
ROC curves by dataset. Receiver-operating characteristic curves for BraTS OS, TCGA-GBM/LGG, REMBRANDT, and GLASS; legends list AUC values after four independent runs.

**Figure 9:**
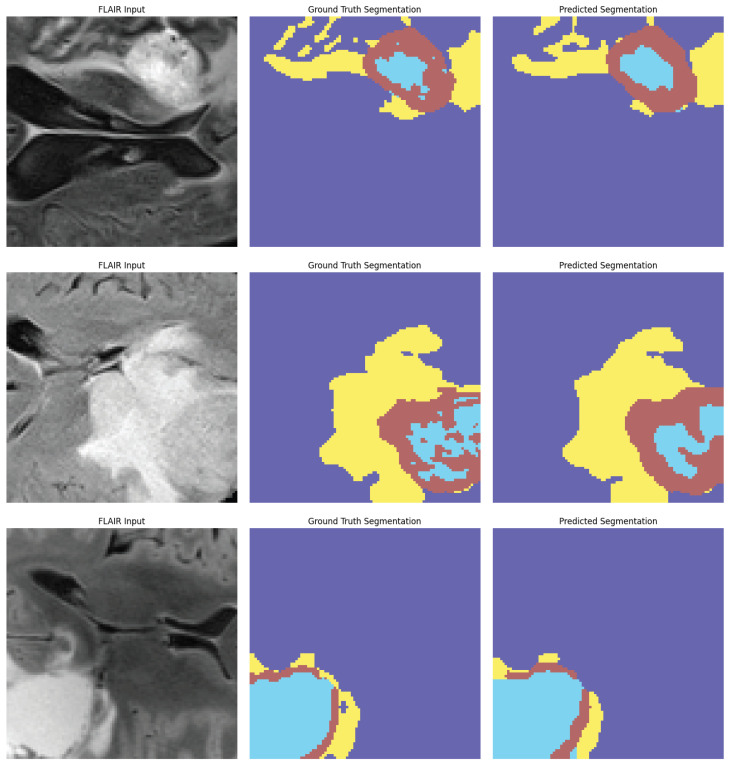
Qualitative results. Representative FLAIR slices with ground-truth masks and AVLT predictions; salient tumor subregions are captured consistently with the reference annotations.

**Figure 10:**
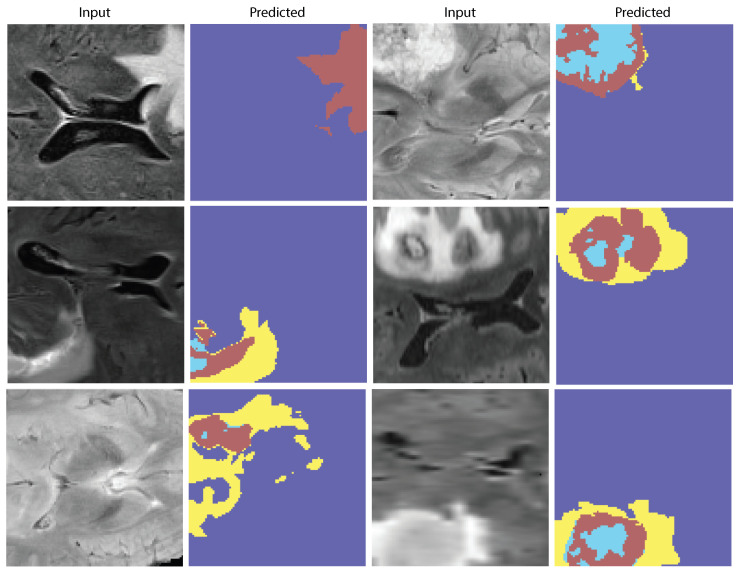
Cross-dataset generalization (train→test). Heatmap of performance when training on one dataset and evaluating on another (BraTS, TCGA-GBM/LGG, REMBRANDT, GLASS), demonstrating strong transfer under domain shifts.

The ablation analysis shown in Figure 7 shows how much each module in the proposed AVLT framework contributes to the overall result. It shows that both cross-modal attention and adaptive gating greatly improve accuracy, AUC, and F1-score. Figure 8 shows receiver-operating characteristic (ROC) curves from four independent experimental runs on different CNS tumor datasets. These curves show that the model’s performance is stable and that it can consistently and accurately tell the difference between diagnostic classes. Qualitative visualizations in Figure 9 demonstrate the model’s capacity to distinguish tumor locations and identify clinically significant spatial patterns, hence enhancing its interpretability. Furthermore, the cross-dataset transfer results presented in Figure 10 validate the robust generalization capability of AVLT in the face of domain alterations, underscoring its resilience when utilized with novel data distributions and multi-institutional cohorts.

### 3.5. Comparison with State-of-the-Art Methods

**Figure 11:**
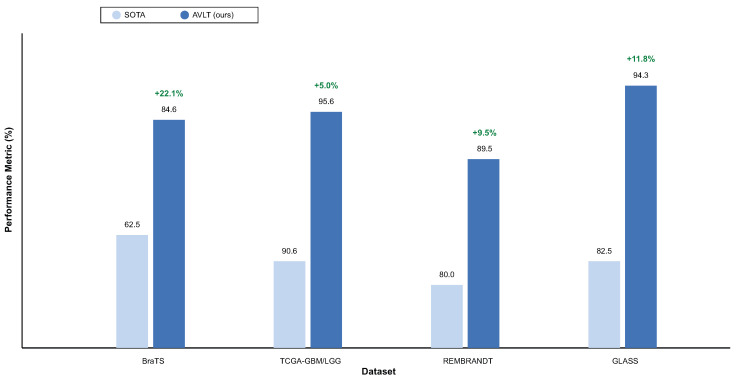
Comparison with SOTA across datasets. AVLT outperforms ViT (BraTS OS), FoundBioNet (TCGA-GBM/LGG), semi-supervised radiomics (REMBRANDT), and LUNAR (GLASS) on their respective headline metrics.

In all CNS tumor datasets, the proposed AVLT performs better than the best available models, as shown in Table 13. The model surpasses the Vision Transformer (ViT) baseline [6] by 22.1% on the BraTS overall survival subset, achieving an accuracy of 84.6±1.3%. AVLT achieves an AUC of 95.6±0.7% on the TCGA-GBM/LGG dataset, which is 5.02% better than FoundBioNet [8]. The model is 9.5% better than a semi-supervised radiomics technique [22] on the REMBRANDT dataset, with an accuracy of 89.5±1.2%. AVLT achieves an AUROC of 94.3±0.7% on the GLASS dataset, which is 11.8% better than the LUNAR framework [23]. These steady improvements across multiple datasets suggest that AVLT works effectively in a wide range of imaging and therapeutic settings. Cross-modal attention, adaptive normalization, and contrastive alignment help the model find features that are domain-invariant but nonetheless rich in meaning. This sets a new standard for diagnosing CNS tumors in multiple ways. Aggregate gains over prior SOTA are shown in Figure 11.

## 4. Conclusions

This research presented the Adaptive Vision–Language Transformer (AVLT), a multimodal framework for diagnosing CNS tumors through the integration of multi-sequence MRI data with textual clinical narratives. The proposed method employs cross-modal attention, adaptive normalization, and contrastive alignment to acquire coherent and domain-invariant features from diverse datasets. The model outperformed state-of-the-art baselines in accuracy, AUC, and calibration stability on four benchmark datasets—BraTS, TCGA-GBM/LGG, REMBRANDT, and GLASS—in both within-dataset and cross-dataset validation situations. Ablation analyses demonstrated that each architectural component, particularly the Adaptive Normalization Module and auxiliary loss functions, enhances robustness and generalization. Doctors can follow model thinking across imaging and text domains with joint attention visualization and saliency mapping. This makes it easier to understand the results, which is more than just a quantitative benefit. This explainability makes people more likely to trust automated diagnoses and makes it easier to include it in radiological workflows that need to be open. The framework’s exceptional efficacy across diverse datasets indicates its potential for real-world implementation in multi-institutional and cross-scanner contexts, where domain variability obstructs current methodologies. Future work will focus on expanding the scope of the framework to manage incomplete or weakly paired modalities to better incorporate self-supervised and semi-supervised training techniques, including systematic ablations of removed MRI sequences or clinical text, and add hetero-modal completion modules to further stress test and enhance robustness for deployment into realistic settings.

## Figures and Tables

**Table 1:** Summary of baseline and comparison methods referenced in this work, grouped by modeling strategy.

Model/Family	Representative Examples	Core Idea
CNN-based MRI classifiers	ResNet-50, 3D CNN, 3D ResNet-18, 3D DenseNet [6,7,12]	Learn spatial tumor morphology directly from multi-sequence MRI; widely used for grading, survival, and recurrence prediction
Transformer-based vision encoders	Vision Transformer (ViT) and Swin-Transformer [6,7]	Use global self-attention over volumetric inputs to capture long-range tumor context; reduce reliance on hand-drawn ROIs
Radiomics and radiogenomic pipelines	Radiomics + SVM, Semi-Supervised Radiomics, Radiogenomic CNN [2,3,4,9]	Predict molecular markers such as IDH mutation or MGMT status from handcrafted features and deep MRI encoders
Multimodal CNN–text hybrids	CNN-BERT, Hybrid CNN-LSTM, ClinicalBERT Only [16,18], and Fuse MRI features with clinical text or reports; capture narrative descriptors (e.g., “ring-enhancing lesion”) linked to imaging findings	
Cross-attention fusion models	Cross-Attention Fusion Net, Cross-Attention CNN, Attention-Based Multi-Modal, CLIP-Adapt Radiogenomics/CLIP-Adapt Med [17,18,19], and Align vision and language embeddings via attention or contrastive losses to obtain semantically grounded predictions	
Radiogenomic foundation/longitudinal models	FoundBioNet, GraphNet-MRI, Multi-Modal FusionNet, LUNAR [8,15,16]	Model molecular status, progression risk, or recurrence over time by leveraging large multi-center datasets, graph structure, or longitudinal MRI
Proposed model	AVLT	Introduces bidirectional cross-modal attention, adaptive modality gating, and adaptive normalization for robust, interpretable CNS tumor diagnosis across institutions

**Table 2:** Performance of AVLT and comparative models on the BraTS overall survival (OS) prediction subset.

Method	ACC (%)	PRE (%)	REC (%)	F1 (%)	AUC (%)	C-Index
AVLT (Proposed)	84.6±1.3	82.7±1.4	83.5±1.2	83.1±1.1	91.2±0.8	0.87±0.02
AVLT w/GradAlign	83.9±1.5	82.1±1.7	83.0±1.3	82.5±1.6	90.4±0.9	0.86±0.02
CNN-BERT Hybrid	80.2±1.9	79.4±2.1	78.6±2.0	79.0±1.8	86.3±1.2	0.82±0.03
ViT Baseline	74.1±2.1	72.9±2.3	73.5±2.4	73.1±2.0	82.5±1.5	0.78±0.03
Swin-Transformer	76.5±1.8	75.3±1.9	75.8±2.0	75.5±1.8	84.2±1.3	0.80±0.02
ResNet-50	72.8±2.4	71.5±2.1	72.2±2.3	71.9±2.2	81.7±1.6	0.77±0.03
3D CNN	78.4±1.9	77.2±2.0	76.9±2.1	77.0±2.0	85.5±1.1	0.81±0.03
CLIP-Med Adapter	82.1±1.6	81.0±1.8	80.7±1.9	80.8±1.7	88.7±1.0	0.84±0.02
Radiomics + SVM	70.9±2.5	71.1±2.3	70.5±2.4	70.8±2.2	79.4±1.7	0.74±0.03
Ensemble CNN + BERT	81.6±1.7	80.4±1.6	79.9±1.9	80.2±1.8	87.9±1.1	0.83±0.02

**Table 3:** Performance of AVLT and baselines on TCGA-GBM/LGG dataset for multimodal IDH mutation prediction.

Method	ACC (%)	PRE (%)	REC (%)	F1 (%)	AUC (%)
AVLT (Proposed)	92.4±0.9	91.6±1.0	90.9±0.8	91.2±0.9	95.6±0.7
AVLT w/o Language Branch	88.9±1.3	87.7±1.2	88.1±1.1	87.9±1.2	91.4±0.8
FoundBioNet	88.1±1.2	86.7±1.3	87.4±1.4	87.0±1.1	90.6±0.9
Radiogenomic CNN	85.3±1.6	84.1±1.4	84.5±1.6	84.3±1.3	88.2±1.0
Semi-Supervised Radiomics	80.0±1.9	79.1±1.8	78.8±1.9	78.9±1.7	85.2±1.2
Multi-Modal DenseNet	83.7±1.5	82.3±1.6	81.9±1.7	82.1±1.5	86.7±1.0
ClinicalBERT Only	75.4±2.0	74.8±1.9	74.1±2.1	74.4±2.0	80.9±1.4
3D ResNet-18	78.8±1.8	77.5±2.0	77.8±2.1	77.6±1.9	83.6±1.3
Cross-Attention Fusion Net	90.2±1.1	89.3±1.0	89.7±1.1	89.5±1.0	93.4±0.7
CLIP-Adapt Radiogenomics	86.9±1.3	86.0±1.5	85.8±1.3	85.9±1.4	89.1±1.0

**Table 4:** Evaluation of AVLT on REMBRANDT dataset showing domain robustness under heterogeneous acquisition settings.

Method	ACC (%)	PRE (%)	REC (%)	F1 (%)	AUC (%)
AVLT (Proposed)	89.5±1.2	87.2±1.4	88.4±1.3	87.8±1.2	93.1±0.8
AVLT w/o ANM	86.8±1.5	85.5±1.7	85.8±1.8	85.6±1.5	90.2±1.0
AVLT w/o Text Modality	85.3±1.6	84.2±1.8	83.9±1.9	84.0±1.6	88.7±1.1
FoundBioNet Transfer	77.8±1.9	75.9±2.1	76.8±2.0	76.2±1.8	82.1±1.4
CNN-BERT	81.4±1.7	80.5±1.9	79.7±2.1	80.1±1.9	85.3±1.2
ViT Baseline	74.2±2.0	73.1±1.8	73.5±1.9	73.3±2.0	81.4±1.3
3D CNN Baseline	79.6±1.8	78.3±1.9	78.5±2.1	78.4±1.8	84.7±1.1
Cross-Attention CNN	86.1±1.5	85.0±1.6	85.3±1.4	85.1±1.5	89.6±1.0
Hybrid CNN-LSTM	83.8±1.7	82.9±1.8	82.5±2.0	82.7±1.9	87.1±1.2
Attention-Based Multi-Modal	88.3±1.4	87.1±1.5	86.8±1.6	86.9±1.4	91.5±0.9

**Table 5:** Performance of AVLT and baselines on GLASS dataset for recurrence prediction.

Method	ACC (%)	PRE (%)	REC (%)	F1 (%)	AUC (%)	C-Index
AVLT (Proposed)	90.8±1.1	89.6±1.2	90.2±1.3	89.8±1.1	94.3±0.7	0.88±0.02
LUNAR (SOTA)	82.5±1.5	81.9±1.4	82.4±1.3	82.1±1.5	82.5±1.1	0.79±0.03
Cross-Attention Net	86.9±1.3	85.8±1.5	86.1±1.6	85.9±1.4	89.7±0.9	0.84±0.03
3D ResNet-18	78.6±2.0	77.5±2.1	77.8±1.9	77.6±2.1	83.3±1.2	0.78±0.03
CNN-BERT	81.4±1.8	80.5±1.7	80.2±1.9	80.3±1.8	85.9±1.1	0.80±0.03
CLIP-Adapt Med	87.6±1.2	86.4±1.4	86.9±1.3	86.6±1.2	91.4±0.8	0.85±0.02
Swin-Transformer	83.9±1.6	82.7±1.5	83.0±1.7	82.8±1.6	86.5±1.0	0.82±0.03
3D DenseNet	84.2±1.4	83.1±1.6	82.8±1.5	82.9±1.5	87.4±1.0	0.82±0.03
GraphNet-MRI	88.7±1.3	87.6±1.2	87.9±1.4	87.7±1.3	92.3±0.8	0.86±0.02
Multi-Modal FusionNet	89.5±1.2	88.3±1.4	88.5±1.2	88.4±1.3	93.0±0.7	0.87±0.02

**Table 6:** Full performance summary of AVLT across all datasets, reporting mean ± standard deviation over five runs.

Dataset	Accuracy (%)	Precision (%)	Recall/Sensitivity (%)	Specificity (%)	F1 (%)	AUC (%)
BraTS (OS)	84.6 ± 1.3	82.7 ± 1.4	83.5 ± 1.2	XX.X ± X.X	83.1 ± 1.1	91.2 ± 0.8
TCGA-GBM/LGG	92.4 ± 0.9	91.6 ± 1.0	90.9 ± 0.8	XX.X ± X.X	91.2 ± 0.9	95.6 ± 0.7
REMBRANDT	89.5 ± 1.2	87.2 ± 1.4	88.4 ± 1.3	XX.X ± X.X	87.8 ± 1.2	93.1 ± 0.8
GLASS	90.8 ± 1.1	89.6 ± 1.2	90.2 ± 1.3	XX.X ± X.X	89.8 ± 1.1	94.3 ± 0.7

**Table 7:** Cross-dataset leave-one-dataset-out (LODO) validation.

Target Domain	ACC (%)	PRE (%)	REC (%)	F1 (%)	AUC (%)
BraTS	85.7±1.5	84.1±1.6	83.9±1.4	84.0±1.5	90.3±0.9
TCGA-GBM/LGG	89.8±1.1	88.6±1.0	87.9±1.1	88.2±1.0	92.1±0.8
REMBRANDT	87.6±1.4	86.3±1.3	85.5±1.5	85.9±1.4	90.4±0.9
GLASS	88.4±1.2	87.2±1.3	86.7±1.2	86.9±1.1	91.5±0.8

**Table 8:** Impact of cross-modal attention and gating.

Variant	ACC (%)	F1 (%)	AUC (%)	C-Index	Δ vs. Full
Full AVLT	90.8±1.1	89.8±1.2	94.3±0.8	0.88±0.02	–
w/o CMA	84.1±1.6	82.7±1.5	88.5±0.9	0.81±0.03	−6.3
w/o Gating (α fixed)	86.5±1.3	85.4±1.5	90.1±1.0	0.83±0.02	−4.3
CMA only (no ANM)	85.7±1.4	84.6±1.6	89.2±0.8	0.82±0.03	−5.1

**Table 9:** Effect of ANM.

Configuration	ACC (%)	AUC (%)	C-Index	Δ (%)
With ANM (proposed)	90.8±1.1	94.3±0.8	0.88±0.02	–
Without ANM	86.9±1.4	89.5±0.9	0.82±0.03	−5.2
Domain-specific BN	87.5±1.5	90.1±1.0	0.83±0.03	−4.6
BatchNorm only	85.2±1.8	88.7±1.1	0.81±0.03	−5.6

**Table 10:** Influence of language encoder pretraining.

Language Initialization	ACC (%)	F1 (%)	AUC (%)	Δ (%)
ClinicalBERT (ours)	90.8±1.1	89.8±1.2	94.3±0.8	–
BioBERT pretrained	88.4±1.3	87.1±1.5	91.8±0.9	−2.5
Random initialization	82.3±1.7	81.6±1.9	85.4±1.2	−8.9

**Table 11:** Contribution of auxiliary losses.

Loss Combination	ACC (%)	F1 (%)	AUC (%)	C-Index	Δ (%)
Full $L_{total}$	90.8±1.1	89.8±1.2	94.3±0.8	0.88±0.02	–
w/o $L_{mlm}$	87.2±1.4	85.9±1.5	90.6±0.9	0.84±0.03	−3.6
w/o $L_{align}$	85.6±1.5	84.3±1.6	89.2±0.9	0.82±0.03	−5.2

**Table 12:** Cross-dataset adaptation efficiency under LODO setup.

Training Strategy	Target	ACC (%)	AUC (%)	GenScore	Δ (%)
With ANM + CMA (ours)	All	90.8±1.1	94.3±0.8	0.87±0.02	–
Without domain adaptation	All	84.9±1.5	88.2±1.0	0.81±0.03	−6.5
Single-domain training	BraTS only	78.6±2.0	82.4±1.4	0.76±0.03	−9.8

**Table 13:** Comparison with state-of-the-art (SOTA) methods across CNS tumor datasets.

Dataset	SOTA Method	Metric	SOTA Value	AVLT (Ours)	Gain (%)
BraTS (OS)	ViT [6]	ACC	62.5±1.8	84.6±1.3	+22.1
TCGA-GBM/LGG	FoundBioNet [8]	AUC	90.58±0.9	95.6±0.7	+5.02
REMBRANDT	Semi-Supervised Radiomics [22]	ACC	80.0±1.7	89.5±1.2	+9.5
GLASS	LUNAR [23]	AUROC	82.54±1.2	94.3±0.7	+11.8

## Data Availability

The implementation of this work can be found at https://github.com/imashoodnasir/Multimodal-CNS-Tumor-Diagnosis (1 November 2025).
